# The paracrine effects of human induced pluripotent stem cells promote bone-like structures via the upregulation of BMP expression in a mouse ectopic model

**DOI:** 10.1038/s41598-018-35546-6

**Published:** 2018-11-20

**Authors:** Karim Oudina, Joseph Paquet, Adrien Moya, Emmanuelle Massourides, Morad Bensidhoum, Nathanaël Larochette, Mickael Deschepper, Christian Pinset, Hervé Petite

**Affiliations:** 1 Laboratory of Bioengineering and Bioimaging for Osteo-Articular tissues, CNRS, UMR, 7052 Paris, France; 2 University Paris Diderot, Sorbonne Paris Cité, Paris, France; 3 I-STEM, CECS, 2 rue Henri Desbruères, 91100 Corbeil-Essonnes, France

## Abstract

Use of human induced pluripotent stem cells (h-iPSCs) for bone tissue engineering is most appealing, because h-iPSCs are an inexhaustible source of osteocompetent cells. The present study investigated the contribution of undifferentiated h-iPSCs and elucidated aspects of the underlying mechanism(s) of the involvement of these cells to new bone formation. Implantation of undifferentiated h-iPSCs seeded on coral particles in ectopic sites of mice resulted in expression of osteocalcin and DMP-1, and in mineral content similar to that of the murine bone. The number of the implanted h-iPSCs decreased with time and disappeared by 30 days post-implantation. In contrast, expression of the murine osteogenic genes at day 15 and 30 post-implantation provided, for the first time, evidence that the implanted h-iPSCs affected the observed outcomes via paracrine mechanisms. Supporting evidence was provided because supernatant conditioned media from h-iPSCs (h-iPSC CM), promoted the osteogenic differentiation of human mesenchymal stem cells (h-MSCs) *in vitro*. Specifically, h-iPSC CM induced upregulation of the BMP-2, BMP-4 and BMP-6 genes, and promoted mineralization of the extracellular matrix. Given the current interest in the use of h-iPSCs for regenerative medicine applications, our study contributes new insights into aspects of the mechanism underlying the bone promoting capability of h-iPSCs.

## Introduction

Availability of stem cells, and the potential of inducing them towards the osteogenic lineage, are motivating the exploration and development of custom-tailored, cell-containing implants known as “bioengineered bone constructs”^1,2^. Such approaches are promising alternatives to physiological limitation, for example, the fact that endogenous regenerative mechanisms do not suffice to repair extensive segmental long-bone defects in humans. Primary adult multipotent stromal cells, derived from bone marrow (BM-MSCs), are the most preferred cell source for stem-cell-based bone regeneration purposes. When addressing the underlying mechanism (s) for the contribution of these cells to bone repair, it was initially thought that BM-MSCs directly participated in osteogenesis *via* their differentiation into bone forming cells^3^. Recent published data, however, provided evidence for an alternative mechanism in which BM-MSCs release several immunomodulatory agents plus trophic factors, which are subsequently involved in regenerative processes^4–6^. Despite the encouraging results reported for the repair of long bones of clinically-relevant volumes in large animals using these cells^7–10^, use of BM-MSCs for tissue repair and tissue engineering applications has several limitations including the following: (i) the therapeutic effectiveness of BM-MSCs is not yet comparable to that of autologous bone grafts^8^; (ii) the proliferation and differentiation capacities of BM-MSCs decline with age, significantly affecting their therapeutic potential^11–14^ and (iii) their long-term expansion in culture may also affect the phenotype of these cells^12^.

An alternative approach aiming at alleviating the drawbacks of BM-MSCs and enhancing the bone forming ability of cell-containing constructs is to substitute pluripotent stem cells for BM-MSCs in these implants. These cells opened new avenues in the field of regenerative medicine because they have an unlimited capacity of self-renewal and can be induced to differentiate into various cell types present in adult mammals (for review^15^). A development of great promise in this field is the work of Takahashi and Yamanaka^16^ who derived novel pluripotent cells by introducing select transcription factors, specifically, C-MYC, POU5F1 (OCT3/4), SOX-2, and KLF4, into somatic cells^16,17^. These cells, known as “induced pluripotent stem cells “(iPSCs), have properties similar to those of embryonic stem cells including the capability to propagate indefinitely, to give rise to every other cell type in the human body, and, specifically to differentiate *in vitro* into the osteoblastic lineage^18–20^. Most importantly, obtaining and using iPSCs are neither subject of ethical concerns (since they are derived from somatic tissues) nor activate immune rejection (because they are genetically tailored to individual patients).

In this study, we hypothesized that human iPSCs (h-iPSCs) loaded onto an osteoconductive scaffold would form new bone. Towards validation of this hypothesis, we assessed the osteogenic capability of h-iPSCs in a mouse ectopic model and observed a positive effect of h-iPSCs on new bone formation. We subsequently analyzed the *in vivo* fate of these cells and found their rapid disappearence post-implantation. To reconcile these apparently paradoxal observations, we hypothesized that h-iPSCs promote new bone formation *via* paracrine effects. We sought, therefore, to establish whether conditioned media from h-iPSCs exhibited osteoinductive effects using cell-based-functional assays. Identification of the mediators responsible for the observed iPSCs biological functions was achieved using biochemical analyses at the molecular level.

## Results

### Characterization of h-iPSCs

As recently described^21^ h-iPSCs generated from human adult myoblast were used. Twenty days after reprogrammation was initiated, compact colony formation of h-iPSCs on feeders with defined edges, morphology characteristics of pluripotent stem cells, and expressing alkaline phosphatase were observed (Supplemental Data section Fig. S1, Frame A and B). Karyotyping (g-banding) revealed a normal karyotype of h-iPSCs (Supplemental Data section Fig. S1, Frame C). When analyzed by flow cytometry, 85% h-iPSCs were positive for the TRA 1-81 and SSEA4 pluripotency markers (Supplemental Data section Fig. S1, Frame D). Quantitative RT-PCR provided evidence that the h-iPSCs VAX1024 exhibited upregulation of the pluripotency markers SOX2, endogenous DNMT3B, and POU5F1 but downregulation of the C-MYC, POU5F1, SOX-2, and KLF-4 transgenes (Supplemental Data section Fig. S1, Frame E and F). Ten weeks after the h-iPSCs VAX1024 graft into the quadriceps of rat, teratomas were formed, exhibiting all three embryonic germ layers (Supplemental Data section Fig. S1, Frames G to K). Taken together, these results demonstrate that newly derived h- iPSCs closely resemble undifferentiated human embryonic stem cells.

In addition, h-iPSCs differentiated towards the osteogenic lineage when cultured in osteogenic medium as evidenced by upregulation of the osteogenic genes, RunX2, ALP, BSP, OC (Supplemental Data section Fig. S2A) and calcium-containing mineral accumulation in the extracellular matrix at day 14 and 21 of culture (Supplemental Data section Fig. S2B).

### Bone-formation induced by h-iPSCs in a mouse ectopic model

**Figure 1:**
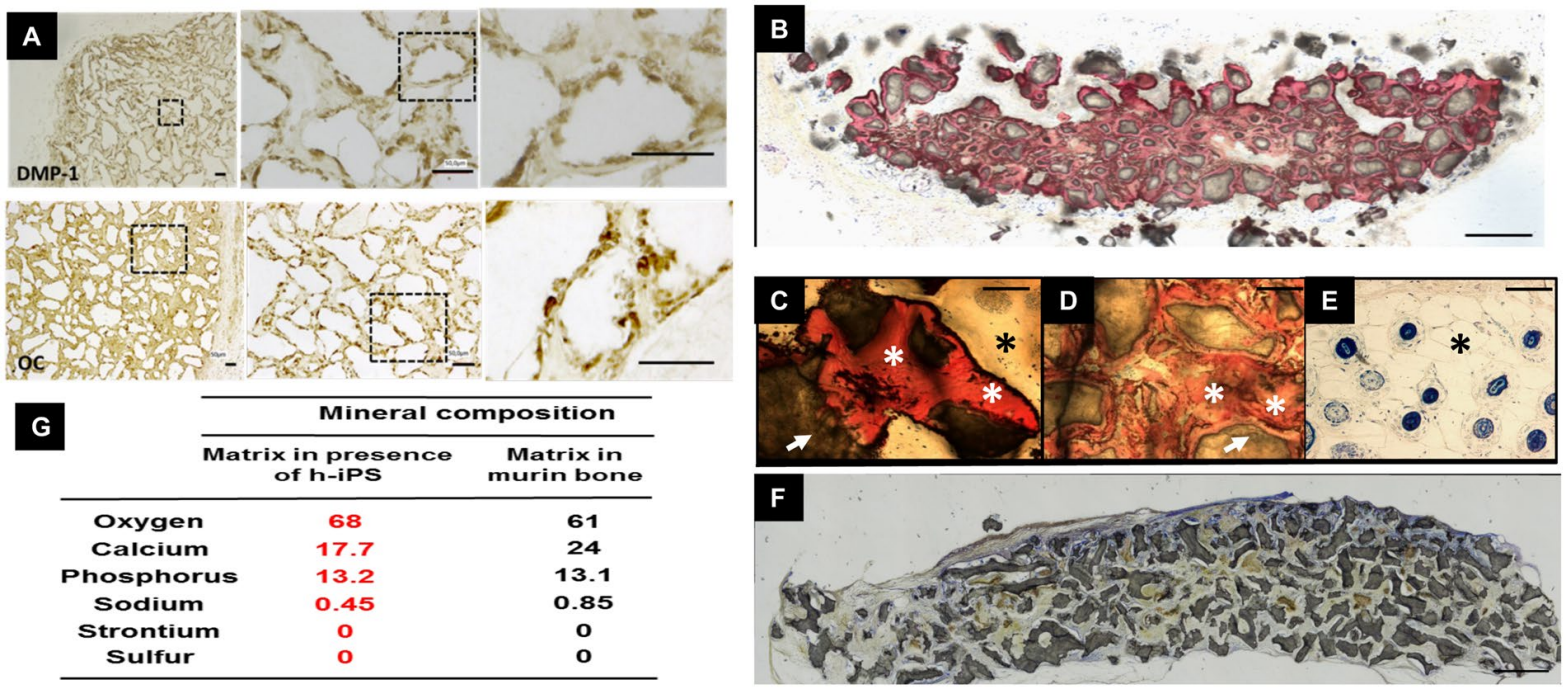
*In vivo*, human induced pluripotent stem cells (h-iPSCs) induced calcium-containing tissue. (**A**) Immuno-histological staining for Dentin matrix acidic phosphoprotein 1 (DMP-1) and Osteocalcin (OC) revealed cell differentiation in the excised implants which had been implanted subcutaneously in mice at day 30 post-implantation. The micrographs on the center and right columns are magnifications of the outlined regions on the respective micrographs and on the respective left column and row. Scale bar = 500 µm. (**B–F**) *In vivo*, h-iPSCs on coral scaffolds induced a mineralized matrix observed after two-month ectopic implantation in mice. Bone formation was visualized on corals containing h-iPSCs (200 µm CSP + 2.10^6^ h-iPSCs; (**B–D**)). In contrast, new bone formation was not observed in the explants of 2 × 10^6^ h-iPSCs without scaffolds (**E**) as well as in cell-free CSP (**F**). Stain: Stevenel blue/picrofuschine (**B**–**F**). The bone-like matrix is stained red (white asterisk); coral particles are brown/black (white arrow); connective tissue is white/pale yellow (black asterisk). Scale bar = 500 µm (**B**,**F**). Scale bar = 100 µm (**C–E**).

The *in vivo* osteogenic capability of h-iPSCs was assessed by implanting cell-containing coral scaffold particles (CSP), cell-free CSP as well as 2 × 10^6^ h-iPSCs without scaffolds subcutaneously into 8-week-old NIH*-*Lyst^bg-J^Foxn1^nu^Btk^xid^ mice. At 1 month post-implantation, immunohistologic examination revealed that the cell-containing CSP explants were positive for both DMP-1 (a marker for mineralizing osteocytes) and osteocalcin (a marker for the bone-forming process; Fig. 1A).

At 2 months post-implantation, light macroscopy examination revealed that the excised constructs were vascularized (data not shown). At that time, histological analyses revealed bone-like structures in all cell-seeded scaffolds (Fig. 1, Frames B to D). No disorganized tissue masses with recognizable tissue types, indicative of teratomas, were observed. To better characterize the mineral phase of the newly-formed bone, scanning electron microscopy (SEM) in the backscattered energy mode (BSE) was used and revealed that the mineral content of the bone-like matrix present in the h-iPSCs-containing CSP was similar, albeit not identical, to the bone matrix of healthy mice (Fig. 1G). In contrast, no significant new bone formation was observed in cell-free CSP (Fig. 1E). as well as in the explants of 2 × 10^6^ h-iPSCs (Fig. 1F). without scaffolds at 2 months post-implantation.

### Origin of the bone-like structures

**Figure 2:**
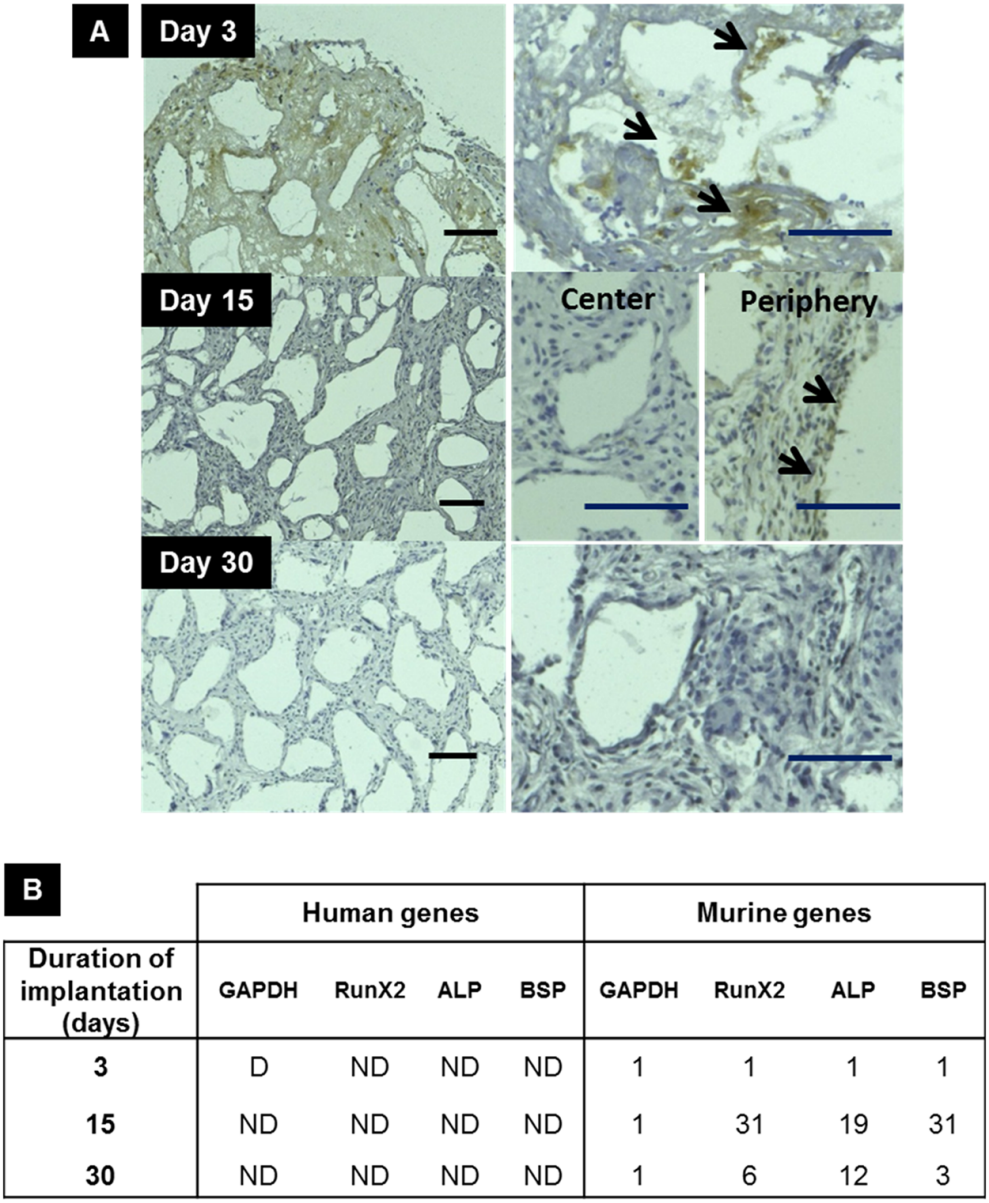
Origin of calcified tissue. (**A**) Progressive disappearance of human cells 3 days post implantation was observed by immuno-histological staining using beta 2 microglobulin human monoclonal antibody. The black arrows point to h-iPSCs. Scale bar = 100 µm in the left and right columns. (**B**) qRT-PCR analysis of the human-specific GAPDH gene provided evidence for the absence of human cells 3 days post implantation. For the human genes: D = Detected. ND = Not Detected. For the murine genes, the numbers indicate fold-increase relative to the expression of murine-specific GAPDH gene at the same time. Abbreviation: GAPDH, glyceraldehyde-3-phosphate dehydrogenase; RunX2, Runt-related transcription factor-2; ALP, Alkaline phosphatase; BSP, Bone sialoprotein.

In order to determine the origin of the observed bone-like structures, which may originate from either h-iPSCs or host osteocompetent cells recruited from the tissues surrounding the implants, the tested h-iPSCs were tracked into the tissue constructs by immunolabeling them with a mouse monoclonal antibody against human ß2-microglobulin at 3, 15 and 30 days post-implantation (Fig. 2). A day 3, a strong immunolabeled h-iPSCs signal was detected in all the implants of interest to the present study (Fig. 2, Frame A Day 3). At day 15, such immunolabelling was reduced or absent in the core of the implants but persisted at time at the implant periphery (Fig. 2, Frame A Day 15). At day 30, faint immunolabelling in localized spots was detectable in only one out of three explants (Fig. 2, Frame A Day 30).

To further investigate the origin of the observed bone-like structures, additional explants were collected at days 3, 15 and 30 post-implantation, mRNA were extracted, and RT-PCR/Q-PCR of human and mouse GAPDH genes were performed. Species-specific RNA detection probes that target the GAPDH housekeeping gene was first verified using total RNA extracted from murine calvaria cells and from h-MSCs. The results of these experiments showed no-cross-species hybridization (data not shown). Analyses of the explanted, cell-containing scaffolds revealed that GADPH mRNA of human origin was detected at day 3 but was undetectable at days 15 and 30 post-implantation (Fig. 2B); these results confirmed rapid disappearance of the h-iPSCs seeded in the implanted cell-containing CSP.

### Assessment of the pro-osteogenic potential of the h-iPSC CM

In addition to contribution to osteogenesis *via* direct differentiation, h-iPSCs may contribute to new bone formation *via* indirect paracrine mechanisms. In order to investigate this alternative, conditioned media from h-iPSCs (h-iPSC CM) was prepared, collected, and its pro-osteoinductive potential was assessed.

#### *The h-iPSC CM exhibited pro-osteogenic ability*

**Figure 3:**
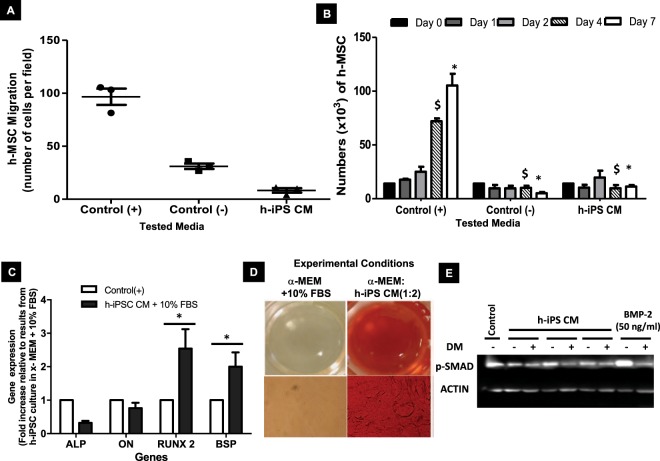
Supernatant conditioned-media from h-iPSCs **(**h-iPSC CM) is osteoinductive. (**A**) Numbers of h-MSC per field which had migrated after 6 hours of exposure to h-iPSC CM. Control(−) = α-MEM only; Control(+) = α-MEM + 10% FBS; h-iPSC CM = α-MEM: h-iPSC CM (1:2). (**B**) Time course of h-MSC proliferation in the presence of h-iPSC CM (Control(+) is significantly higher than the two others conditions at day 4 ($) and day 7 (*), n = 3; p < 0.01, Bonferroni post tests). (**C**) Expression of ALP, Osteonectin (ON), RunX2 and BSP mRNA in h-MSC cultured *in vitro* in either α-MEM + 10% FBS or α-MEM:CM h-iPSC tested media for 21 consecutive days (n = 9; p < 0.05, Bonferroni posts tests). (**D**) After 21 days of cell culture, calcium-containing mineral was present in the extracellular matrix of the h-MSCs exposed to h-iPSC CM. Stain: Alizarin Red. Magnification: upper row = X2; lower row = X10. (**E**) Phosphorylation of SMAD 1/5/8 (p-SMAD) was detected by immunoblot after pretreatment (or not, DM-) of C2C12 with 4 µM dorsomorphin (DM+) for 30 min and subsequent treatment with either BMP-2 or h-iPSC CM for 30 min. Actin was used as the loading control. Abbreviation: h-MSC, human mesenchymal stem cell; SMAD, small mothers against decapentaplegic; BMP, bone morphogenetic protein.

To assess the effect of h-iPSC CM on select cell functions, the capability of h-iPSC CM to promote migration, as well as to induce proliferation and differentiation of h-MSCs, were assessed using assays pertinent to each aforementioned cell function.

h-MSCs exposed to h-iPSC CM exhibited no chemotaxis after one day (Fig. 3A). In addition, there was no h-MSC proliferation under h-iPSC CM compared to h-MSCs exposed to α-MEM supplemented with 10% FBS at all time points tested (Fig. 3B).

In contrast, h-MSCs cultured in the presence of α-MEM supplemented with 10% FBS for 21 days did not differentiate (Fig. 3, Frame C white bars), whereas h-MSCs exposed to h-iPSC CM for the same time period expressed osteogenesis-related genes including RunX2 and BSP (Fig. 3, Frame C black bars) and had calcium-containing deposits in their extracellular matrix stained with Alizarin Red (Fig. 3, Frame D right column).

#### *The h-iPSC CM expressed BMP-like activity*

**Figure 4:**
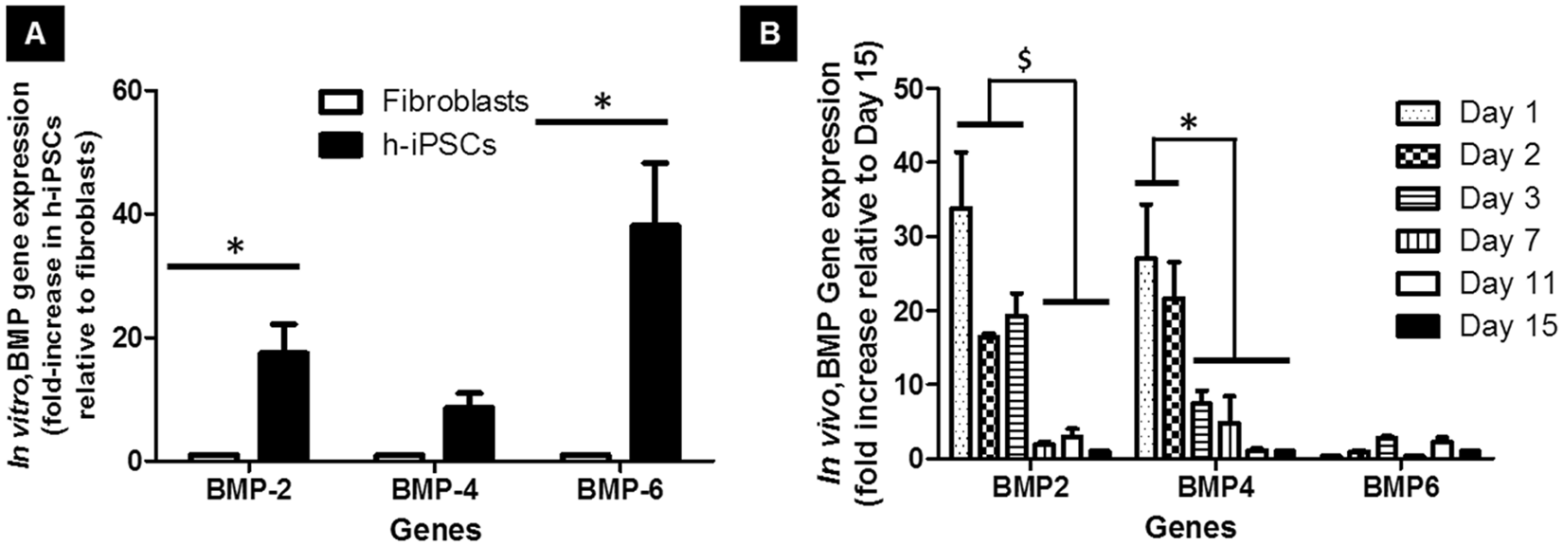
Human induced pluripotent stem cells (h-iPSCs) expressed BMPs. (**A**) Comparison of BMP-2, BMP-4 and BMP-6 mRNA expression by h-iPSCs and fibroblasts cultured *in vitro* for 3 consecutive days (n = 3; p < 0.01; Bonferroni post tests). (**B**) Time course of the relative mRNA level of BMP-2, BMP-4 and BMP-6 (as compared to that of the same gene at day 15) expressed by h-iPSCs loaded on coral scaffolds and implanted ectopically in mice (n = 6; p < 0.01, Bonferroni post tests). Abbreviation: BMP, bone morphogenetic protein.

To investigate whether the osteoinductive capability of h-iPSC CM may be explained by the release and bioactivity of BMPs, phosphorylation of the downstream effectors SMAD1/5/8, was monitored after exposure of h-iPSC CM to C2C12 in either the presence or absence of dorsomorphin, a selective inhibitor of BMP type I receptors^22^. Phosphorylation of SMAD 1/5/8 occurred when C2C12 were exposed to h-iPSC CM. In contrast, C2C12 pre-incubated with dorsomorphin and then treated with h-iPSC CM exhibited no phosphorylation of SMAD 1/5/8 (Fig. 3E). To further investigate which BMP was involved, expression of BMP-2, BMP-4 and BMP-6 in h-iPSCs and fibroblasts was assessed; a 17-, 8- and 37-fold increase in the expression of BMP-2, BMP-4 and BMP-6, respectively, was obtained in h-iPSCs compared to the respective results obtained using human fibroblasts (Fig. 4A). Last, but not least, quantification of BMP-2 by ELISA revealed that h-iPSC CM contained 40 pg BMP-2 per 10^6^ h-iPSCs over a 24 hours cell culture period (Supplemental Data section Fig. S3).

To further gauge the importance of BMPs in the underlying bone induction mechanisms, the expression of human BMP-2, BMP-4 and BMP-6 *in vivo* was determined during the first fifteen days post-implantation of h-iPSCs in mice. A progressive decrease in the relative expression of BMP-2 and BMP-4 (as compared to that of the same gene at day 15) was observed (Fig. 4B). Most importantly, these BMPs were detected at significant level (CT values below 35 cycles) in the explants at day 1 and 2. In contrast, the expression of BMP-6 was detected with CT values above 35 cycles at these time points.

## Discussion

Implants containing cells, especially stem cells, are very promising alternative treatments for bone repair. To date, such approaches are limited because of cell-related issues, for example, the limited proliferative capacity of BM-MSCs *in vivo*. A promising, but yet not fully tapped alternative, is the use of h-iPSCs. These cells can be propagated indefinitely, while still retaining the capacity to differentiate into all cell types in the body of mammals, and are a potentially inexhaustible supply of human osteocompetent cells.

In the present study, we focused on bone regeneration using undifferentiated h-iPSCs *in vivo*. We demonstrated for the first time that, when seeded onto osteoconductive scaffolds and implanted ectopically in mice, undifferentiated h-iPSCs promoted the formation of bone-like structures of murine origin with little, if any, direct contribution by h-iPSCs. Most importantly, we demonstrated for the first time that h-iPSCs participated in the observed formation of bone-like structures *via* the release of osteoinductive factors including BMPs.

Literature reports have established that pre-differentiated h-iPSCs loaded onto osteoconductive scaffolds promoted new bone formation in ectopic and orthotopic sites in mice^19,23–25^. The present study significantly expanded the scope of previous studies by showing, for the first time, that undifferentiated, muscle-derived h-iPSCs loaded onto osteoconductive scaffolds induced the formation of bone-like structures in the ectopic mouse model. The presence of exogenous cells was indispensable for bone formation because scaffolds alone did not induce new bone formation. In this regard, the results of the present study differ with those reported in the literature for adipose-derived h-iPSCs, which required both osteoconductive and osteoinductive cues to promote bone formation when implanted into an exoskeletal subcutaneous site in mice^26^. This difference in results implies, but does not prove definitively, that the tissue origin of reprogrammed cells may play a significant role in the expression of their osteogenic ability. In this respect, several studies reported that the epigenetic memory of the tissue of origin of iPSCs may affect the function of these cells pertinent to new tissue formation (for review^27–29^).

Rapid disappearance of the transplanted, undifferentiated, muscle-derived h-iPSCs was observed using immunohistochemistry to track the cells of human origin in the mice tested in the present study. These data were further substantiated by quantitative species-specific detection of the GAPDH housekeeping gene using the qRT-PCR method. Our data are in agreement with published reports that the number of undifferentiated murine^30^, rat^31^, and sheep^32^ MSCs decreased within the first days post-implantation (albeit at a more gradual rate) when loaded into ceramic scaffolds and implanted in an ectopic mouse model. Moreover, using the qRT-PCR method for quantitative species-specific detection of GAPDH, no human cells were detected, in the liver, kidney, brain, lungs and spleen 30 days post implantation (data not shown). These results suggest that, most probably, the observed rapid disappearance of h-iPSCs resulted from early cell death rather than outward systemic migration of h-iPSCs towards other organs (data not shown). Consistent with this interpretation are the results of a study reported in the literature which established the irremediable death of h-MSCs which had been labeled with Luc-GFP, distributed within fibrin gel/coral scaffolds, and implanted subcutaneously in nude mice^33^. The reasons for the early death of h-iPSCs are unknown and need further investigation. It is reasonable, however, to speculate that, the early h-iPSC death is similar to the trend reported for MSCs^30,34^, and is the result of multifactorial aspects that may include oxidative stress, inflammation, release of cytotoxic cytokines^35^, and ischemia^33^ as well as nutrient depletion^36,37^.

Paradoxically, the rapid disappearance of implanted, undifferentiated, muscle-derived h-iPSCs was associated with a positive effect on new bone formation. To reconcile these apparently contradictory trends, we investigated the indirect paracrine role of transplanted h-iPSCs on new bone formation. Selected pro-osteoinductive effects of h-iPSC CM were, therefore, assessed using h-MSCs in *in vitro* assays. First, h-iPSC CM had no effect on the migration and proliferation of h-MSCs (Fig. 3A,B). In contrast, exposure of h-MSCs to h-iPSC CM for 21 days resulted in a 1.85-fold mRNA upregulation of RunX2 (a transcription factor involved in osteoblastic differentiation and skeletal morphogenesis) and of Bone Sialoprotein (a component of the bone extracellular matrix) (Fig. 3C). Moreover, exposure of h-MSCs to h-iPSC CM led to calcium accumulation in the extracellular matrix after 21 days of cell culture (Fig. 3D). Taken together, these findings are indicative of the osteoinductive bioactivity of the chemical content of h-iPSC CM.

The observation that h-iPSC CM was osteoinductive raised the question of the molecular origin of this bioactivity. Because some members of the BMP family are osteoinductive, we investigated whether h-iPSCs released BMP compounds, monitored phosphorylation of their downstream effectors SMAD 1/5/8, and observed activation of BMP-SMAD 1/5/8 signaling (Fig. 3E). Along the same lines, a 17-, 8- and 37-fold expression of the BMP-2, BMP-4 and BMP-6 mRNA levels, respectively, was observed in h-iPSCs when compared to human fibroblasts after 3 days in culture (Fig. 4A). These results are in agreement with the reported high expression of BMP-2, BMP-3, BMP-5, and BMP-6 in osteogenic mouse iPSCs when compared to mouse MSCs^20^. Last, but not the least, the BMP-2 protein was present in h-iPSC CM in detectable amount specifically, 40 pg of BMP-2 per 10^6^ h-iPSCs after 24 hours. These results were substantiated by the detection of BMP-2 and BMP-4 at significant level (CT values below 35 cycles) in the cell-containing constructs at day 1 and day 2 post-implantation (Fig. 4B).

In the present study, undifferentiated muscle-derived h-iPSCs loaded onto coral scaffolds implanted in mice formed bone-like structures which were not obtained when the same cells were placed into fibrin hydrogels and injected ectopically in the mouse model. In other words, the sole provision of an osteoconductive cue was a necessary and sufficient condition for promoting the bone forming capability of undifferentiated, muscle-derived h-iPSCs. Coral scaffolds release calcium upon their resorption^38^. Given that extracellular calcium promotes bone formation from bone marrow MSCs by amplifying the effects of BMP-2 on SMAD signaling^39^, it is tempting to speculate that calcium signaling also plays also a crucial role in the h-IPSC-mediated osteogenesis.

Coral scaffolds were chosen as the osteoconductive cue because it is an established delivery vehicle for MSCs in clinically-pertinent animal models^7,8^ but may not be the optimal choice considering recent advances in the development of nanostructured scaffolds exhibiting relevant biochemical and biophysical cues^40,41^. Considering the paracrine effects of bioactive compounds released by h-iPSCs, it would be interesting to investigate whether scaffolds composed of these novel material formulations may improve the osteoinductive potential of h-iPSCs either via enhanced BMPs release from these cells or via improved delivery of secreted BMPs which affect functions of the host, bone-forming cells pertinent to new tissue formation.

In conclusion, the present study demonstrated that h-iPSCs induce new bone formation *via* paracrine pathways that include the release of BMPs. Looking forward, and given the current wide interest in the use of h-iPSCs for regenerative medicine applications, the present study contributes new insights into aspects of the mechanism underlying the bone promoting capability of h-iPSCs; such knowledge can be applied in developing improved methodologies in order to enhance release of BMPs by h-iPSCs *in vivo*.

## Materials and Methods

### h-iPSCs

Human adult myoblasts (VAX1024) from an healthy patient, with signed informed consent, were provided by Celogos laboratory (Paris, France). Myoblast preparation was done according to patent US2010/018873 A1. h-iPSCs were derived from the VAX1024 myoblasts, by transferring C-MYC, POU5F1, SOX-2, and KLF-4 transgenes using retroviral vectors as described by Massourides^21^. The h-iPSCs were maintained in mTeSR™1 medium (STEMCELL Technologies) on Matrigel-coated plates (Corning). Details of h-iPSC cell culture, and characterization are given in the Supplemental Data section.

### Human mesenchymal stem cells (h-MSCs)

The tissues were collected with the respective patient’s consent in agreement with Lariboisiere Hospital regulations after acceptance from the Institutional Review Board -IRB 00006477- of HUPNVS, Paris 7 University, AP-HP. Bone marrow was obtained as discarded tissue during routine bone surgery from 5 adult donors. h-MSCs were isolated from the bone marrow of each patient using a procedure adapted from literature reports^42^, characterized^43^, pooled at an equal ratio at passage 1, and were cultured in α-MEM (Dutscher, Brumath, France) supplemented with 10% Fetal Bovine Serum (FBS), that is, a humidified, 37 °C, 5% CO_2_, 95% air environment. Cells at passage 4–5 were used for the experiments.

### Coral scaffolds

The exoskeleton of natural coral (*Porites species*)^44^ used in the present study was provided by Biocoral France (La Garenne Colombes, France). These scaffolds contained particles of different sizes and they will be referred to, thereafter, as 80–200 µm coral scaffold particles (CSP).

### Preparation of cell-seeded scaffolds

Aliquots (each 40 mg) of sterile coral particles^45^ were placed immersed in mTeSR™1 medium at 37 °C for at least 1 hour. At that time, the supernatant was removed, 2.10^6^ h-iPSCs were re-suspended in 1 ml mTeSR™1, seeded onto the coral particles, and allowed to adhere for 90 min. The supernatant culture medium was removed and first 100 µL of 18 mg/mL human fibrinogen (Tissucol, Baxter), and then 10 µL thrombin (500 IU/mL; Tissucol; Baxter) were added to form cell- and CSP-containing hydrogel constructs. Cell-seeded constructs were transferred in 5 ml of mTeSR™1 and were maintained under standard cell-culture conditions overnight. Cell-free constructs were also separately embedded in fibrin gels and used as controls.

### Animals

NIH-Lyst^bg^ Foxn1^nu^ Btk^xid^ mice (8-week-old females; 20–22 g, Charles River, Boston, USA) were used. The animal-related protocols for these studies had received approval by the local Ethics Committee on Animal Research of Lariboisiere (number CEEAL V/2010-01-04), and were carried out in accordance with the European Guidelines for Care and Use of Laboratory Animals (EEC Directives 86/609/CEE of 24.11.1986).

### Surgical Procedures

Pre-anaesthetic medication, anesthesia and surgery were performed as described previously^46^. Briefly, incisions were made along the vertebral axis of each animal and separate subcutaneous pouches were created by blunt dissection. The pouches were randomly assigned to 3 groups: Group 1: 80–200 μm constructs seeded with 2 × 10^6^ h-iPSCs (n = 3); Group 2: 2 × 10^6^ h-iPSCs without a scaffold (n = 3); Group 3: 80–200 μm cell-free coral scaffolds only (n = 3); Group 2 and Group 3 were used as controls. Eight weeks post-implantation, the explants were fixed and processed for undecalcified histology. Regions of newly-formed bone were visualized using light microscopy on sections stained by Stevenel blue/picrofuschine and quantified using NIS element BR software (Nikon). The extent of new bone formation on each section examined was expressed as a percentage of the total area of the implant.

### Scanning electron microscopy with backscatter electron (SEM-BSE) imaging

The mineral composition of the bone matrix present in the thin sections of the excised implants was examined using SEM-BSE imaging following established techniques which had been described previously^47^.

### RT-PCR analysis

For *in vitro* samples, the cells in culture were rinsed with PBS, scraped and the collected pellets were frozen at −80 °C until processing. For *in vivo* samples, excised, cell-containing constructs were collected and frozen at −80 °C until processing.

Quantitative RT-PCR were performed using the TaqMan® gene expression assay and an iCycler thermocycling apparatus (MyiQ^TM^, Bio-Rad). All reagents were purchased from Applied Biosystems. Each RT-PCR analysis was performed in triplicate using 25 ng cDNA each time.

To identify the origin of cells in the implanted constructs, specific amplifications of the human GAPDH and human/mice 18S cDNA were performed using RT-PCR. No cross-reactivity between human and murine cDNA was observed when amplification of human GAPDH was performed using the specific TaqMan® gene expression assay.

Extracted mRNAs were analyzed using RT-PCR targeting murine osteogenic differentiation markers, specifically, Runt-related transcription factor-2 (RunX2), Alkaline phosphatase (ALP), Bone sialoprotein (BSP), Osteonectin (ON) and Osteocalcin (OC) and human osteogenic differentiation markers, specifically, RunX2, ALP, BSP, OC, BMP-2, BMP-4 and BMP-6. The sequences of oligonucleotide primers used for the real-time PCR are listed in the Supplemental Data section, Table S1.

### Immuno-histochemical analysis

All explants (n = 3 per group) were fixed in 10% formalin, decalcified in 18% EDTA, and embedded in paraffin. Tissue sections (each 5–15 µm) were cut using a rotary microtome, deparaffinized and rehydrated. Some of these sections were stained using hematoxylin and eosin (to detect teratomas). Immunohistochemical labeling was performed using either a ß2-microglobulin mouse monoclonal antibody (specific for the human-origin ß2-microglobulin), or Dentin matrix acidic phosphoprotein 1 (DMP-1, Santa Cruz Biotechnology) or OC (Abcam); followed by counter-staining using hematoxylin according to manufacturer’s instructions.

### Identification of the origin of cells in the implanted constructs

To determine the origin of the cells present in the implanted constructs at various time point postimplantation, 2.10^6^ h-iPSCs were loaded on 80–200 μm CPS and were implanted ectopically in mice. After 3, 15 and 30 days post-implantation, the mice were sacrificed using barbiturate overdoses (Dolethal®, Vetoquinol, France) and the implants were excised. The human GAPDH cDNA present in each explant was quantified using RT-PCR (n = 6 for each time point) and the human cells were detected on histological sections using a mouse monoclonal antibody specific for ß2-microglobulin of human origin (n = 3 for each time point) as described in the “RT-PCR analysis” and “Immuno-histochemical analysis” sections.

### Preparation of supernatant conditioned-media from h-iPSCs

For preparation of conditioned media (CM), the h-iPSCs were seeded at 25.000 cells/cm^2^ in individual wells of 24-well cell-culture plasticware and were cultured in α-MEM medium (PAN Biotech; USA) containing 5 g/L glucose but no added serum in a humidified, 37 °C, 5% CO_2_, 95% N_2_ and 0.1% oxygen environment for 3 days. The low oxygen tension was chosen to best mimic the ischemic milieu to which h-iPSCs are exposed upon implantation. At the prescribed time, the supernatant CM was collected, aliquoted, and thereafter kept at −80 °C. The supernatant CM from h-iPSC will be referred to as “h-iPSC CM” in the rest of this manuscript.

### mRNA expression of osteoinductive cytokines released by h-iPSCs in the h-iPSC CM

h-iPSCs and h-MSCs (used as control) were seeded at 25.000 cells/cm^2^ in 24-well cell-culture plasticware and were cultured in α-MEM medium (PAN Biotech; USA) containing 5 g/L glucose without any added serum in a humidified, 37 °C, 5% CO_2_, 95% N_2_ and 0.1% oxygen environment for 3 days. All experiments were performed in triplicate at three separate occasions.

### Chemotaxis of h-MSCs exposed to h-iPSC CM

The chemo-attractive potential of the chemical compounds contained in h-iPSC CM was determined using the Boyden chamber cell migration assay. Briefly, 600 µL of h-iPSC CM were placed in the bottom well and 15 × 10^3^ h-MSCs were seeded on the top of the 8-μm diameter porous membrane (coated with 0.5% gelatin) of the transwell. After 6 hours, only the cells that had migrated to the bottom of the porous membrane were fixed *in situ*, stained using Hematoxylin Eosine, and counted using light microscopy (magnification: 10X).

### Proliferation of the hMSCs exposed to h-iPSC CM

For this purpose, 10^5^ h-MSCs were seeded in each well of 12-well cell-culture plasticware and cultured in either (i) α-MEM, (ii) α-MEM supplemented with 10% FBS, or (iii) a medium containing α-MEM:h-iPSC CM (1:2) without any added serum. Cell were counted at different days. These experiments were performed in triplicate at one occasion.

### Osteogenic differentiation of h-MSCs exposed to h-iPSC CM

10^5^ h-MSCs were seeded in 12-well cell-culture plasticware and cultured in either (i) α-MEM supplemented with 10% FBS, (ii) medium containing α-MEM:h-iPSC CM (1:2), or (iii) osteogenic differentiation medium (Lonza, USA). At different days of culture, cell mRNA was extracted and analyzed using real-time quantitative RT-PCR targeting select human, osteogenic-differentiation markers. The presence of calcium-containing deposits in the extracellular matrix was assessed using Alizarin Red stain.

### Immunoblot analysis of BMP–responsive SMAD phosphorylation

The studies of SMAD activation were performed as previously described^22^. Briefly, 3 × 10^5^ C2C12 cells (cell line with osteogenic capability) were seeded into 6-well plate and cultured in Basal medium Eagle (BME) with 10% FBS (24 h), and were then cultured in BME without FBS overnight. Cells were then either pre-incubated or not, with dorsomorphin (D+) for 30 min, followed by the addition of either h-iPSC CM (n = 3) or recombinant BMP-2 (R&D Systems) for one hour.

The cells were mechanically homogenized using RIPA buffer, separated by SDS-PAGE, immunoblotted with antiphospho-SMAD1/5/8 (Cell Signaling) or anti α-tubulin(Upstate/Millipore) antibodies, and visualized using a chemiluminescent assay (Lumi-Light; Roche; Germany). The immunoreactive proteins present in these samples were visualized and quantified using IVIS Lumina imager (Xenogen).

### Statistical Analysis

Results are expressed as the mean ± standard deviation (SD). Statistical differences between sets of data were determined using Bonferroni post-test. Differences with p < 0.05 were considered significant.

## Electronic supplementary material

Suppl.information
