# Supplementary material for: The paracrine effects of human induced pluripotent stem cells promote bone-like structures via the upregulation of BMP expression in a mouse ectopic model
Source: Sci Rep. 2018 Nov 20;8:17106. doi: 10.1038/s41598-018-35546-6 (PMC6244408; doi:10.1038/s41598-018-35546-6)
Supplement: Supplementary file 1 — Suppl.information [file 41598_2018_35546_MOESM1_ESM.docx]

***The paracrine effects of human induced pluripotent stem cells promote bone-like structures via the upregulation of BMP expression in a mouse ectopic model***

**Running title**: h-IPSCs promote bone-like structures

**Authors**: Karim OUDINA^1,2^, Joseph PAQUET ^1,2^, Adrien MOYA^1,2^, Emmanuelle MASSOURIDES ^3^, Morad BENSIDHOUM ^1,2^, Nathanaël LAROCHETTE ^1,2^, Mickael DESCHEPPER ^1,2^, Christian PINSET ^3^, Hervé PETITE ^1,2^

- ^1^ Laboratory of Bioengineering and Bioimaging for Osteo-Articular tissues, CNRS, UMR 7052, Paris, France
- ^2^ University Paris Diderot, Sorbonne Paris Cité, Paris, France

^3^ I-STEM, CECS, 2 rue Henri Desbruères, 91100 Corbeil-Essonnes, France

**Supplemental Data**

For characterization of h-iPSCs VAX1024, we performed karyotyping analysis by g-banding method, teratomas formation, alkaline phosphatase activity (Sigma), qPCR for pluripotency markers and transgenes expression analysis.

**Karyotyping**

Conventional cytogenetic analyse, g-banding, was performed on h-iPSCs VAX1024 as previously described by Varela (Varela et al. 2012).

**Teratomas formation:**

After being included in pure matrigel, seven million of h-iPSC single cells were grafted inside quadriceps of Nude rats of 5 weeks old. After 10 weeks, rats were euthanized and teratomas fixed in 10% Formaldehyde. Paraffin inclusion, hematoxylin/eosin staining and teratomas analysis were done. All rats were bought from Charles River Laboratories and housed at I-Stem. All animal experimentations were performed in compliance with European ethical guidelines (86/609/EEC).

**mRNA Purification and qRT-PCR**

Total RNA was extracted and purified according to the RNeasy Mini Kit protocol (Qiagen).

RNA level and quality were checked using the Nanodrop technology. For each sample, 500 ng of total RNA were reverse transcribed with random primers (Life technologies), oligo(dT) (Fermentas) and dNTP (Life technologies) using Superscript III Reverse Transcriptase (Life technologies).

For quantitative PCR, cDNA was amplified using primers listed in **supplemental table S2.** Detection was performed using an Applied 7900HT system (Applied SDS version 2.3). The relative RNA level was calculated using 2-ΔΔCt with Cyclophilin A as housekeeping gene.

The expression of pluripotency (Pouf5, Sox2 and DNMT3B) genes was performed using the embryonal carcinoma line "2102Ep" as a reference sample (Fig S1, Frame E). The expression of transgenes (Pouf5, Sox2, C-MYC and KLF-4) was carried out using fibroblasts at day 4 after retrovirus exposition as a reference sample (Fig S1, Frame F).

**Osteogenic differentiation of h-iPSCs**

h-iPSC (10^5^ cells) were seeded in each well of 12-well cell-culture plastic ware and cultured in either DMEM F12 containing 10% FBS or in LONZA® osteogenic differentiation medium (Lonza,Walkersville, USA). After 7, 14 and 21 days of culture, cell mRNA was extracted and analyzed using real-time quantitative RT-PCR targeting select human, osteogenic-differentiation markers, specifically, RunX2, ALP, BSP, and OC.

At day 21, the presence of calcium-containing deposits in the extracellular matrix of the cultured cells was assessed using Alizarin Red stain and by examining pertinent specimens using light microscopy (magnification: 10X).

**BMP-2 concentration in h-iPSC-CM.**

Elisa assay (Duoset ELISA # DY355 R&D systems) was performed to determine the amount of BMP-2 released in the h-iPSC-CM. To this aim, h-iPSCs were seeded at 4.000 cells/cm2 in T25cm2 cell-culture plasticware and were cultured in α-MEM medium (PAN Biotech; USA) containing 5 g/L glucose but no added serum in a humidified, 37°C, 5% CO2, 21 % oxygen environment for 3, 6 and 24 hours (n=2).

**Reference**

Varela C, Denis JA, Polentes J, et al., Recurrent genomic instability of chromosome 1q in neural derivatives of human embryonic stem cells. The Journal of Clinical Investigation, 2012; 122 (2).

**Figure S1: Characterization of h-iPSCs VAX1024.** Alkaline phosphatase activity was observed on either the entire petri dish area (Frame A) or in a single cell colony of h-iPSCs VAX1024 (Frame B; scale bar=200 µm). G-banding analysis of h-iPSCs VAX1024 showed a normal karyotype (Frame C). Flow cytometry analysis revealed that, 85% h-iPSCs were positive for the TRA 1-81 and SSEA4 pluripotency markers (Frame D). Quantitative RT-PCR showed that the h-iPSCs VAX1024 exhibited (i) an upregulation of the pluripotency markers DNMT3B, endogenous SOX2 endogenous, and POU5F1 endogenous (Frame E, results are express as a relative quantification using EC cells as gene level reference) and (ii) a downregulation of the C-MYC, POU5F1, SOX-2, and KLF-4 transgenes (Frame F, results are express as a relative quantification using fibroblast at day 4 as gene level reference). Teratomas were obtained with h-iPSCs VAX1024 after intra-quadricep injections into nude rats (Frame G). Histological examination revealed that the h-iPSCs VAX1024 had differentiated into all three germ layers (Frame H; scale bar=1,000 µm). Select regions of the teratoma histology (Frame H, black arrow) are magnified to illustrate formation of the following: ectoderm (Frame I); mesoderm (Frame J); and endoderm (Frame K). Frame I, J and K; scale bar= 60 µm.

**Figure S2. h-iPSCs differentiated toward the osteogenic lineage**. Frame A: Time course expression of RunX2, ALP, BSP, OC and Nanog mRNA by the h-iPSCs cultured *in vitro* in either DMEM F12 containing 10% FBS or LONZA® osteogenic medium for 21 consecutive days (n=3). Frame B: At 7, 14 and 21 days of culture, the respective extracellular matrices were stained using Alizarin Red to reveal calcium-containing mineral accumulation. Scale bar=100 µm. D=days of culture.

**Figure S3**. **BMP-2 concentration in h-iPSC CM.** Elisa assay was performed to determine the amount of BMP-2 released in the h-iPSC CM. Data are expressed in pg of BMP-2 produce by 10^6^ h-iPSCs during the exposure time.


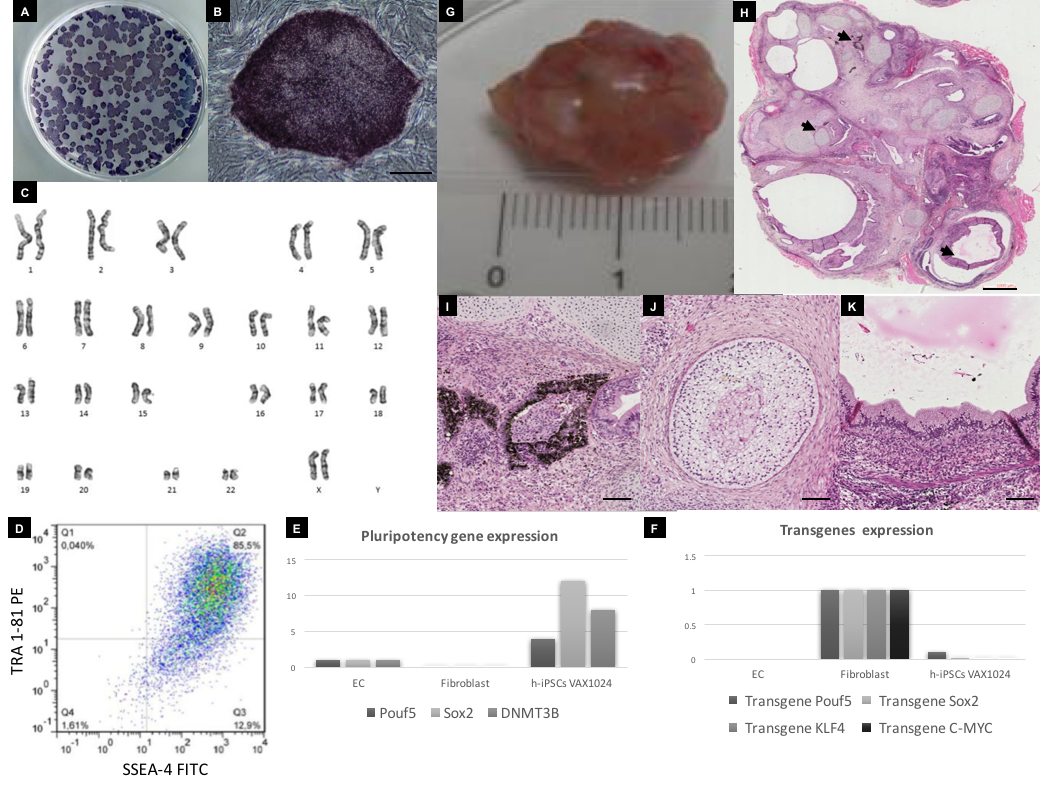


**Figure S1:** Characterization of h-IPSCs.

.


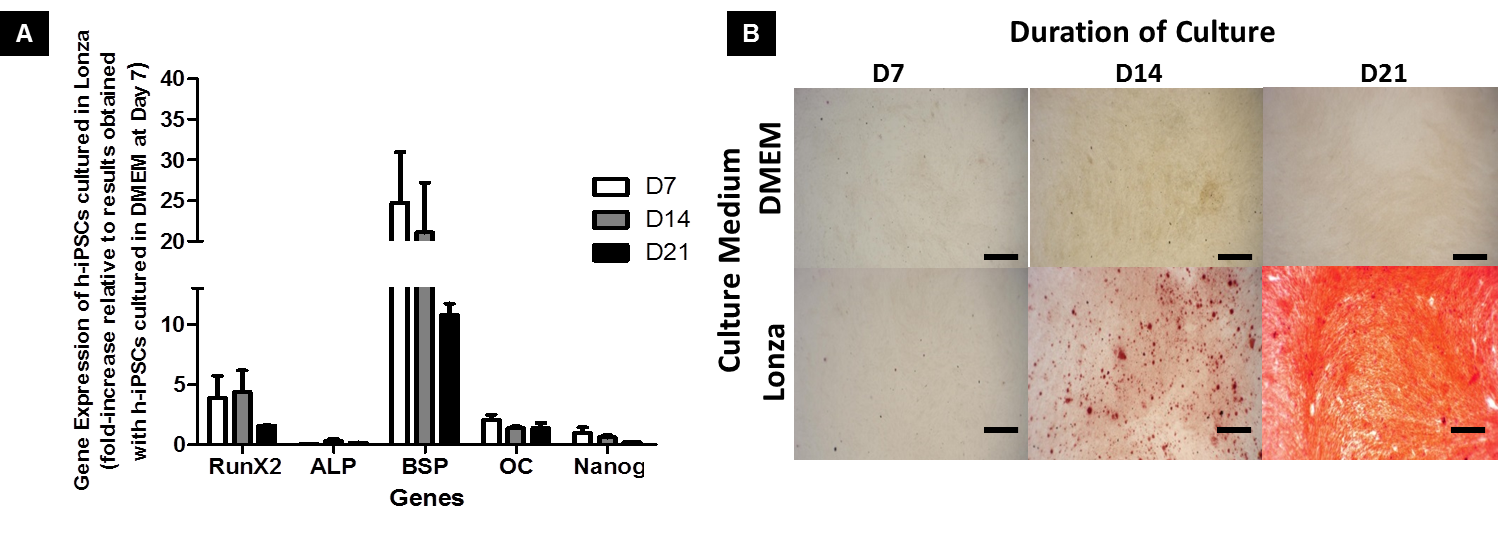


**Figure S2:** h-IPSCs differentiated toward the osteogenic lineage.

**
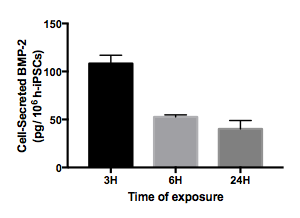
**

**Figure S3:** Amount of BMP-2 in h-iPSC CM.

| **Specie** | **Gene** | **Sequence**  **(Commercial, Applied Biosystems)** |
| --- | --- | --- |
|  | GAPDH  RunX2 | Hs00266705_g1  Hs00231692_m1 |
|  | ALP | Hs01029144_m1 |
|  | BSP | Hs00960942_m1 |
|  | OC | Hs00609452_g1 |
| Human | Nanog | Hs02387400_g1 |
|  | BMP-2 | Hs00154192_m1 |
|  | BMP-4 | Hs00370078_m1 |
|  | BMP-6 | Hs01099594_m1 |
|  | 18s | Hs99999901_s1 |
|  | GAPDH | Mm99999915_g1 |
| Murine | RunX2 | Mm00501580_m1 |
|  | ALP | Mm00475834_m1 |
|  | BSP | Mn00436767_m1 |

**Table S1:** List of primers. Abbreviation: GAPDH, glyceraldehyde-3-phosphate dehydrogenase; RunX2, Runt-related transcription factor-2; ALP, Alkaline phosphatase; BSP, Bone sialoprotein; OC, Osteocalcin; BMP, bone morphogenetic protein.

| Gene product | Primers |
| --- | --- |
| SOX2 endogenous | Fw 5'-CCGGTACGCTCAAAAAGAAA-3' |
|  | Rv 5'-TGTCATTTGCTGTGGGTGAT-3' |
| POU5F1 endogenous | Fw 5'-CCTCACTTCACTGCACTGTA-3' |
|  | Rv 5'-CAGGTTTTCTTTCCCTAGCT-3' |
| POU5F1 endogenous | Fw 5'-CCTCACTTCACTGCACTGTA-3' |
|  | Rv 5'-CAGGTTTTCTTTCCCTAGCT-3' |
| DNMT3B | Fw 5'-ATAAGTCGAAGGTGCGTCGT-3' |
|  | Rv 5'-GGCAACATCTGAAGCCATTT-3' |
| POU5F1 transgene | Fw 5'-CCTCACTTCACTGCACTGTA-3' |
|  | Rv 5'-CCTTGAGGTACCAGAGATCT-3' |
| SOX2 transgene | Fw 5'-CCCAGCAGACTTCACATGT-3' |
|  | Rv 5'-CCTTGAGGTACCAGAGATCT-3' |
| KLF4 transgene | Fw 5'-GATGAACTGACCAGGCACTA-3' |
|  | Rv 5'-CCTTGAGGTACCAGAGATCT-3' |
| MYC transgene | Fw 5'-TGCCTCAAATTGGACTTTGG-3' |
|  | Rv 5'-CGCTCGAGGTTAACGAATT |

**Table S2:** List of primers for pluripotency and transgenes expression
